# Prolonged repeated inseminations trigger a local immune response and accelerate aging of the uterovaginal junction in turkey hens

**DOI:** 10.3389/fphys.2023.1275922

**Published:** 2023-11-22

**Authors:** Sunantha Kosonsiriluk, Kent M. Reed, Sally L. Noll, Ben W. Wileman, Marissa M. Studniski, Kahina S. Boukherroub

**Affiliations:** ^1^ Department of Animal Science, University of Minnesota, Saint Paul, MN, United States; ^2^ Department of Veterinary and Biomedical Sciences, University of Minnesota, Saint Paul, MN, United States; ^3^ Select Genetics, Willmar, MN, United States

**Keywords:** aging, avian, immune response, insemination, reproductive tract, transcriptomics, turkey, uterovaginal junction

## Abstract

Artificial insemination is a standard practice in the turkey breeder industry to ensure the production of fertile eggs. Even though hens are inseminated on a weekly basis, their fertility tends to decline after a few weeks of production. Avian species have a specialized structures called sperm storage tubules (SSTs), located in the uterovaginal junction (UVJ) of the oviduct. The ability of SSTs to store sperm is directly correlated with the fertility of the hen. The objective of the study was to examine changes in the transcriptome of the turkey hen’s UVJ in response to the presence of sperm at three key stages of production. We hypothesized that repeated and prolonged exposure to sperm would alter the transcriptome of the UVJ. Samples were collected from virgin hens prior to the onset of lay, as well as from sham-inseminated (extender only) and semen-inseminated hens at early lay, peak lay, and late lay. Gene expression profiling of the UVJ was examined, and a differential expression analysis was conducted through pairwise comparisons between semen- and sham-inseminated groups at each production stage and across production stages. In the early laying stage, no significant gene expression changes were found between semen- and sham-inseminated groups. However, at peak lay, genes related to lipid biosynthesis, Wnt signaling, cell proliferation, and O-glycan biosynthesis were upregulated in the semen group, while the immune response and cytokine-cytokine receptor interaction were downregulated. In the late lay stage, the transcription pathway was upregulated in the semen group, whereas the translation pathway was downregulated. The local immune response that was suppressed during peak lay was increased at the late laying stage. In the semen-inseminated group, the UVJ exhibited advanced aging at the late laying stage, evidenced by reduced telomere maintenance and translation processes. The results from this study provide valuable insights into the alteration of the UVJ function in response to the presence of sperm at different stages of production and throughout the production cycle. Targeting the modulation of local immune response and addressing aging processes after peak production could potentially prevent or delay the decline in fertility of turkey breeder hens.

## 1 Introduction

Turkey breeder operations heavily rely on artificial insemination as a crucial method for producing fertile eggs that will eventually hatch and give rise to meat-producing turkeys. However, maintaining optimal fertility in turkey hens has proven to be a complex challenge. Hens reach peak production at around 6–8 weeks of lay, then fertility starts to gradually decline. Previous attempts to enhance fertility through increased insemination frequency and varying sperm insemination quantities have yielded minimal improvements (Bakst, 1988). The decline in fertility observed in avian species can be attributed to various factors, including hormonal imbalances, disease and immune-related factors, environmental influences, the timing of insemination, and the selection of sperm within the vaginal tract (Assersohn et al., 2021). One critical aspect affecting fertility is the storage of sperm in the hen’s reproductive tract.

Sperm can be stored in the reproductive tract for several weeks, primarily in tubular invaginations of the surface epithelium at the uterovaginal junction (UVJ) region, known as sperm storage tubules (SSTs) (Holt, 2011). The UVJ plays a pivotal role in sperm selection, and its function is directly correlated with the fertility of avian species. Studies have shown that the number of spermatozoa residing in the SSTs positively correlates with the number of spermatozoa embedded in the perivitelline layer of oviductal eggs, thus improving the fertility of birds (Brillard and Bakst, 1990; Santos et al., 2013). Additionally, high-fertility line hens exhibit more SSTs, higher numbers of stored sperm, and longer sperm storage durations compared to low-fertility line hens, supporting the importance of SSTs in fertility (Brillard et al., 1998; Yang et al., 2021a).

Spermatozoa are considered foreign to the female immune system and promptly stimulate a local immune response in the reproductive tract for elimination. During mating, immune-modulatory genes are expressed in the UVJ of chickens (Atikuzzaman et al., 2015), leading to the recruitment of immune cell populations to the UVJ, potentially impacting sperm survivability (Das et al., 2005). Previous studies have highlighted the critical role of suppressing local immunity in the UVJ against sperm for successful sperm survival and storage in laying hens (Das et al., 2009) and transforming growth factor β (TGFβ) has been implicated in this mechanism (Das et al., 2006).

Beyond the immune-related factors, various other elements contribute to sperm survival and storage in the SSTs of birds. Fatty acids have been found to influence sperm survival, maintenance, and motility in the SSTs (Huang et al., 2016). Additionally, several proteins, including carbonic anhydrase (Holm et al., 1996; Holm et al., 2000), avidin (Foye-Jackson et al., 2011), and aquaporins (Zaniboni and Bakst, 2004) play crucial roles in supporting the function of SSTs. While these findings provide valuable insights into the sperm storage function, there remain many factors that are not fully understood. Further research is needed to comprehensively explore the mechanisms behind sperm storage and its regulation in the UVJ of avian species.

Recent studies have used transcriptomics to explore the sperm storage function of the SSTs in chickens, shedding light on the comprehensive information about sperm storage in the UVJ (Han et al., 2019; Yang et al., 2020; Yang et al., 2021a; Yang et al., 2021b; Yang et al., 2023). However, it is crucial to acknowledge that turkey breeder reproduction is different from commercial chicken layers. The production lifespan of turkey breeder hens is approximately 28 weeks, unlike commercial chicken layers that produce eggs regularly for 2–3 years (El-Sabrout et al., 2022). This difference in the production cycle between the two species may indicate distinct mechanisms of reproductive tract development, maintenance, and regression. Additionally, sperm storage capacity and duration in the SSTs can vary significantly among avian species (Birkhead and Møller, 1992), and there is a substantial difference in the number of SSTs observed between chicken and turkey breeders (Bakst et al., 2010).

Recent transcriptomic studies on turkey focusing on the SSTs have provided valuable insights into the mechanism of SSTs in sperm storage (Brady et al., 2022). However, to gain a comprehensive understanding of the sperm storage process, it is imperative to conduct a transcriptomic study encompassing the entire turkey UVJ tissue. This approach allows for the exploration of integrated functions among various cell populations, including epithelial (both ciliated and non-ciliated), goblet, fibroblasts, muscle, SST, and immune cells located within the UVJ. Gaining insights into how these components work collectively will provide valuable insight into fertility dynamics in turkey breeder hens. In this study, we characterize transcriptomic changes in the turkey UVJ in response to repeated insemination across the production cycle. We hypothesized that prolonged exposure to sperm leads to alterations in the UVJ transcriptome, contributing to a decline in fertility among turkey breeder hens.

## 2 Methods

### 2.1 Experimental animals

Twenty-eight turkey breeder hens (*Meleagris gallopavo*) were generously donated by a turkey breeding company that collaborated with this study. The experimental birds were randomly selected from a parent stock breeder flock. A sample log of experimental birds was maintained to coordinate group assignment. The hens were provided with a conventional diet containing 12% protein during the pre-production phase and 18% protein during egg production. Water was available *ad libitum*. At 29 weeks of age, the hens were photo-stimulated with a light schedule of 14.5 h of light and 9.5 h of darkness. Beginning at 31 weeks of age, the hens were inseminated using 2 million fresh sperm diluted 1:1 with the extender, Turkey A1 Diluent with gentamicin (Southeast Poultry Health Services, Polkton, NC). Raw semen was collected to 16 milliliters (mL) by vacuum suction into 20 mL syringes pre-filled with an 8 mL extender. The viability of sperm was more than 85%, and the motility was 4.5 or greater on a scale of 5. Insemination was carried out three times in the first week and once per week throughout the 27 weeks production lifespan. The sham-inseminated groups were inseminated with the extender, following the same schedule as the flock insemination. Four virgin birds were collected 24 h before the first insemination. Semen- and sham-inseminated birds (*n* = 4/group) were collected at early lay (2 days after the first insemination), peak lay (8 weeks after the first insemination), and late lay (27 weeks after the first insemination). The protocol used in this study was approved by the University of Minnesota Institutional Animal Care and Use Committee.

### 2.2 UVJ tissue collection

Hens were humanely euthanized with assisted mechanical cervical dislocation. The lower reproductive tract was removed and dissected from the uterus to the vaginal opening. The UVJ tissue was dissected and divided horizontally into 1 cm^2^ sections. One tissue section was snap-frozen in liquid nitrogen and stored at −80°C for RNA extraction and another section was fixed in 10% neutral-buffered formalin (NBF) for histology and group confirmation using hematoxylin and eosin (H&E) stain.

### 2.3 Tissue processing and H&E staining

UVJ tissues were fixed overnight at the room temperature in 10% NBF and processed through dehydration, clearing, and infiltration with paraffin using an Epredia STP 120 spin tissue processor (Epredia, Kalamazoo, MI). The tissues were embedded in paraffin blocks, sectioned at 5 μm, and mounted on charged slides (VWR International, Radnor, PA). H&E staining included deparaffinization and rehydration of tissues through a series of 100% xylene, a mixture of 50% xylene and 50% ethanol, 100% ethanol, 75% ethanol, and distilled water. Tissues were stained with hematoxylin, Gill III (Sigma-Aldrich, St. Louis, MO) for 2 min, then the slides were quickly dipped twice in acidic alcohol (0.1% HCl in 75% ethanol) and rinsed with several changes of distilled water until the water became clear. Tissues were immersed in 75% ethanol and stained with eosin for 2 min. Tissues were then dehydrated through a series of 75% ethanol, 100% ethanol, a mixture of 50% xylene and 50% ethanol, and 100% xylene. Tissues were mounted with mounting medium, and coverslips were applied. Tissues were observed under a light microscope to confirm the histology of the UVJ. The presence or absence of sperm in the SST was examined to confirm the assignment of semen- and sham-inseminated groups, respectively.

### 2.4 RNA extraction, cDNA library construction, and sequencing

Total RNA was extracted using the RNeasy Midi Kit and treated with DNase (Qiagen, Germantown, MD, United States) following the manufacturer’s protocol. The samples were submitted to the University of Minnesota Genomics Center for quantity and quality assessment using the RiboGreen assay (Thermo Fisher Scientific, Waltham, MA, United States) and TapeStation 4200 (Agilent, Santa Clara, CA, United States), respectively. Sample RNA integrity numbers ranged from 8.0 to 9.4 and these were processed through cDNA library construction. Indexed libraries were constructed with the TruSeq Stranded mRNA Sample Preparation Kit (Illumina, Inc., San Diego, CA, United States) and size-selected for approximately 200 bp inserts. The libraries were multiplexed and sequenced on the Illumina NovaSeq 6000 platform using 150 PE flow cell to produce 51 bp paired-end reads.

### 2.5 Transcriptomic data analysis

Sequence processing and mapping were conducted as described in Reed et al. (2022) using the turkey genome (UMD 5.1, ENSEMBL Annotation 104). Gene IDs were determined as described in Reed et al. (2022). Raw read counts were uploaded to the integrated Differential Expression and Pathway analysis (iDEP) version 0.96 which is a web-based tool available at http://bioinformatics.sdstate.edu/idep96/ (Ge et al., 2018). In the pre-processing step, data were normalized using the cpm function in edgeR. Genes that were expressed at <0.5 counts per million (CPM) in a library were filtered out. Normalized counts were transformed using edgeR log2(CPM+c), with a pseudo count *c* = 4 added to all counts before transformation. The transformed data were used for exploratory data analysis including clustering and principal component analysis (PCA). K-means clustering analysis was performed using gene expression pattern across samples of the top 2000 most variable genes. Differentially expressed genes (DEGs) were determined through pairwise comparisons between semen- and sham-insemination at early lay, peak lay, and late lay and across production cycle in both groups using DESeq2 (Love et al., 2014). For all comparisons, false discovery rate (FDR) < 0.05 and fold-change > |1.5| were considered statistically significant.

Enrichment analysis was performed using DEGs from each pairwise comparison. The Database for Annotation, Visualization and Integrated Discovery (DAVID) Functional Annotation Tool (Huang et al., 2009; Sherman et al., 2022) was used for gene ontology (GO) analysis and Kyoto Encyclopedia of Genes and Genomes (KEGG) pathway enrichment. The annotation summary results of each comparison were used to create enrichment bubble plots (http://www.bioinformatics.com.cn/srplot). Pathway analysis was performed using Parametric Gene Set Enrichment Analysis (PGSEA) (Jambusaria et al., 2018). KEGG pathway diagrams (Kanehisa et al., 2017) were used to visualize gene expression data using Pathview (Luo and Brouwer, 2013). The transcriptome data have been deposited with the National Center for Biotechnology Information (NCBI) Sequence Read Archive (SRA) database, BioProject ID PRJNA1003625.

## 3 Results

### 3.1 The presence of sperm in the SST of semen inseminated UVJ tissues

**FIGURE 1:**
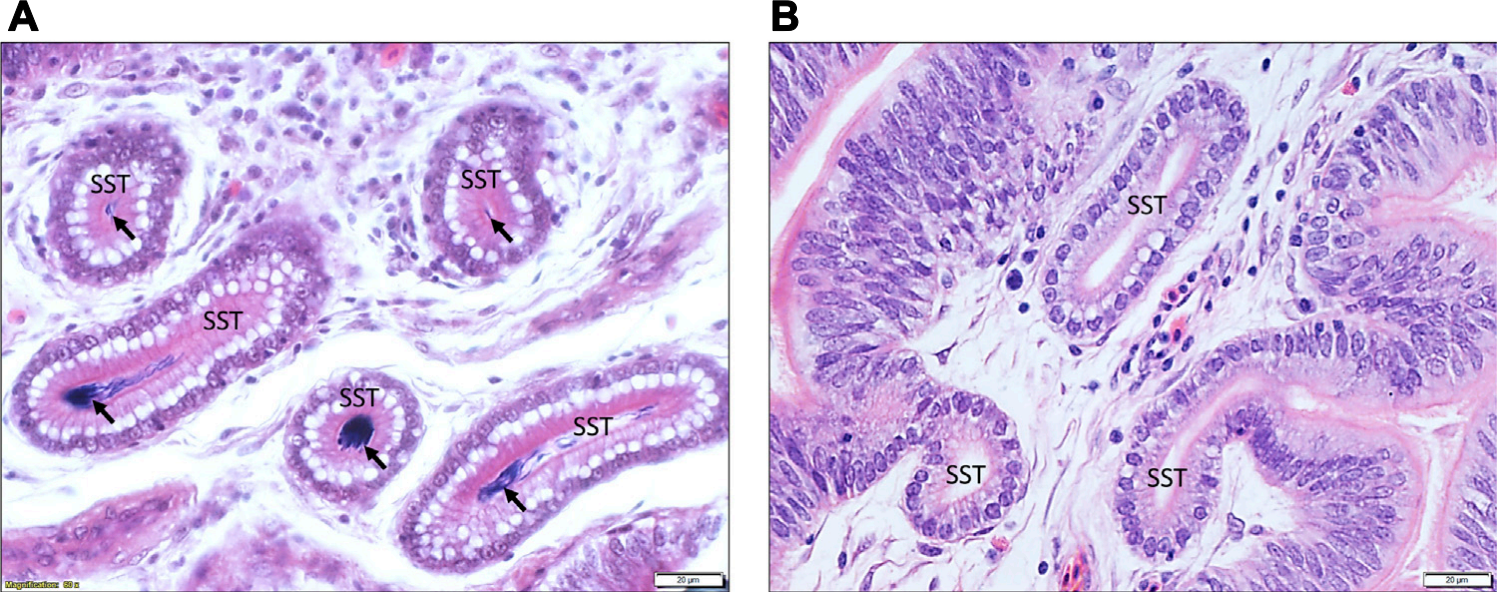
An illustrative example of the histology of the UVJ tissue containing SST stained with hematoxylin and eosin. **(A)** Arrows indicate sperm present in the SST of UVJ tissues from a semen-inseminated bird. **(B)** Sperm are lacking in the SST of UVJ tissues in a sham-inseminated bird.

The histology of UVJ tissues was examined to validate the accuracy of collected samples. The presence of sperm demonstrated successful semen insemination in semen-inseminated groups. The key distinguishing feature of UVJ tissue from the adjacent uterus and vagina tissues is the presence of SSTs. It is noteworthy that SSTs were consistently observed in all samples collected and that the presence of sperm was exclusively found in the SSTs of the semen-inseminated groups of all stages of production. This finding confirms successful tissue collection, semen insemination, and the validity of our group assignments. Figure 1 provides an illustrative example of H&E stained UVJ tissues, showcasing sperm presence in the SSTs from semen-inseminated birds (Figure 1A) and the absence of sperm in the SSTs from sham-inseminated birds (Figure 1B).

### 3.2 Gene expression data clustering according to stages of production

**FIGURE 2:**
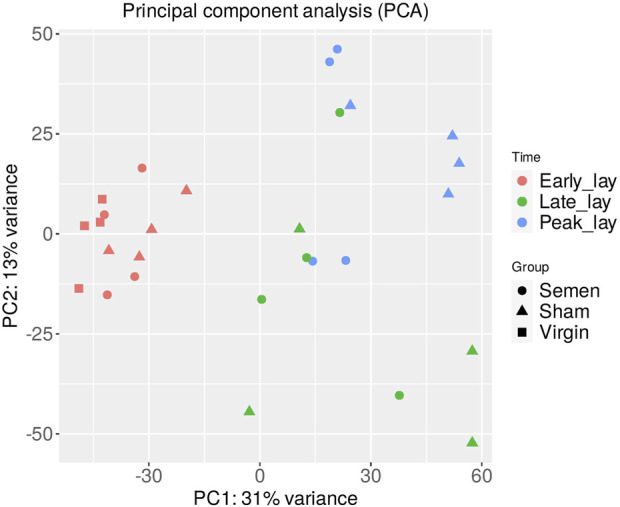
Principal component analysis (PCA) of the UVJ samples. A PCA plot of RNA-seq normalized read counts data shows clusters of samples based on their similarity. Samples with similar characteristics appear close to each other and samples with dissimilar characteristics are farther apart. Groups are denoted by different shapes and sampling times by color.

A total of 750 million paired-end sequence reads were generated across the 28 samples (7 groups × *n* = 4 per group). The average number of reads per library was 26.8 million, with consistently high-quality reads (Q values ranging from 36.1 to 36.2). After filtration, 14,828 genes out of the 17,970 total expressed genes were used for the subsequent PCA. The results of the PCA (Figure 2) revealed distinct clusters representing semen, sham, and virgin samples during early lay, well-separated from the clusters of samples obtained during peak lay and late lay. This striking separation underscores the significant influence of the production stage on the overall gene expression patterns in the UVJ.

**FIGURE 3:**
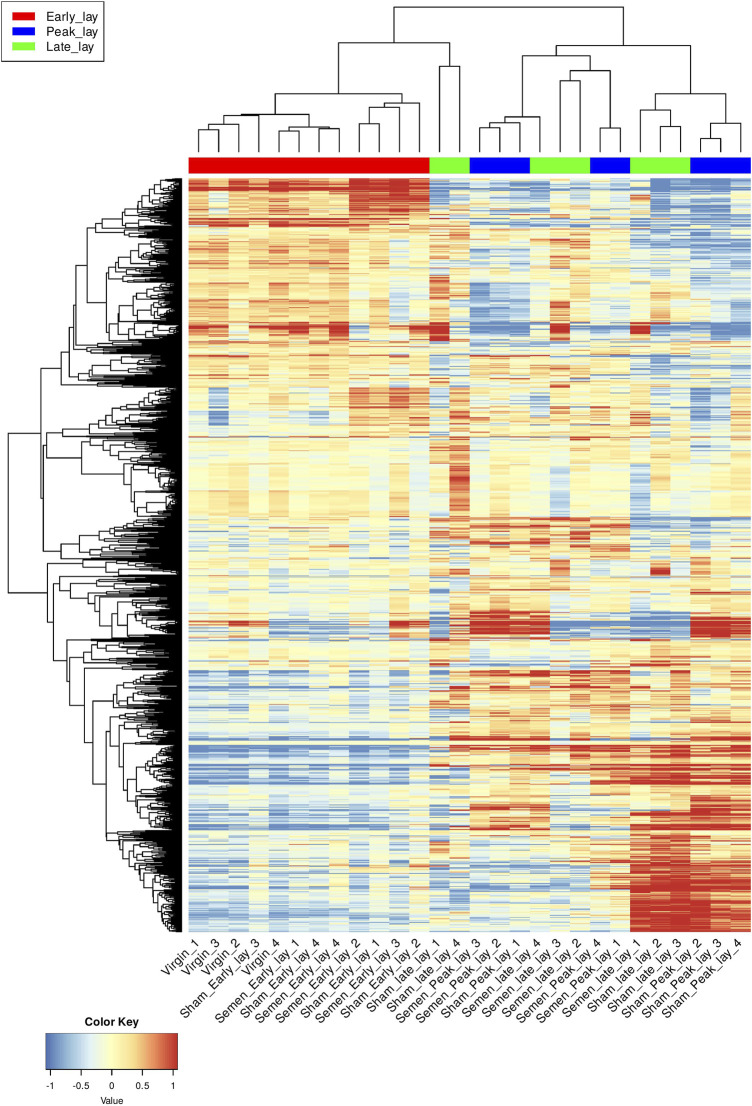
Hierarchical clustering of the UVJ samples. A hierarchical clustering heatmap based on 1,200 most variable genes of transformed data. Each row represents a gene and each column represents a sample. Genes were grouped based on expression level and samples were grouped based on similarity of gene expression profile. The color key indicates gene expression values.

To further explore the dynamic changes in gene expression over the stages of production, we utilized a hierarchical clustering heatmap based on the 1,200 most variable genes (Figure 3). These findings provided insights into the shifts in gene expression profiles as hens entered peak production. Although the clustering of peak lay and late lay samples showed some heterogeneity, the observed variations in gene expression may be indicative of distinct reproductive performances among individual hens.

**FIGURE 4:**
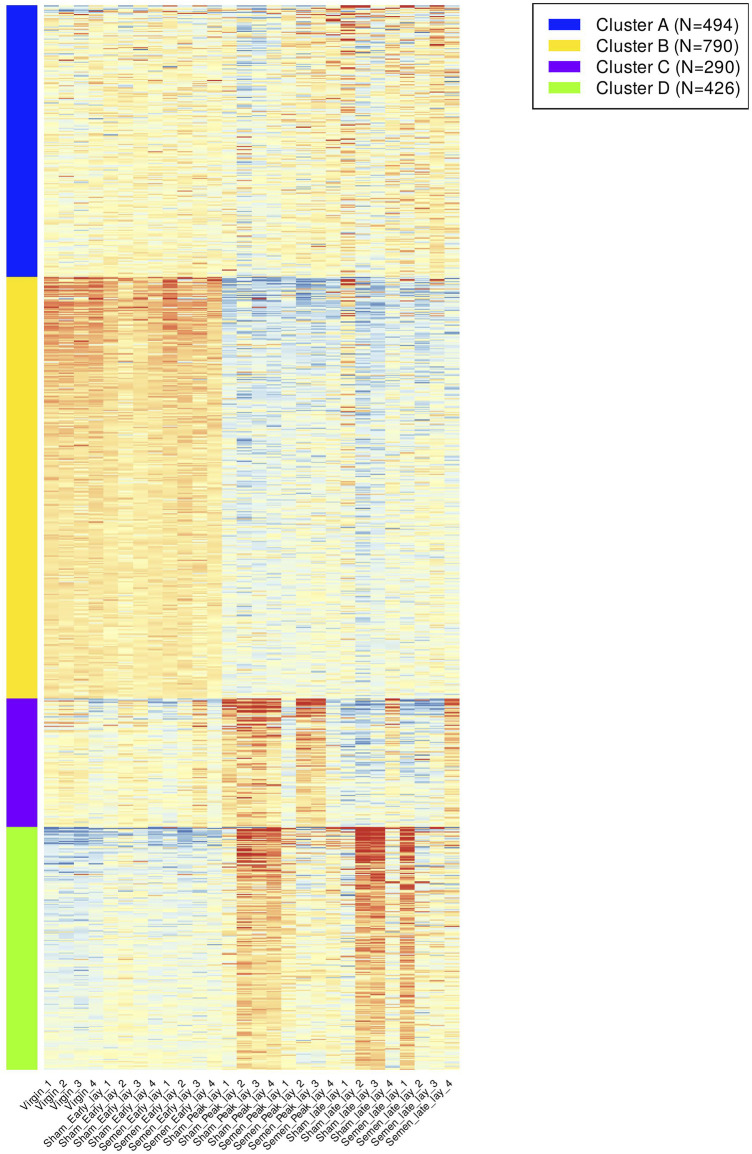
K-means clustering of gene expression pattern and enriched pathways across samples. A heatmap of the top 2,000 most variable genes across all samples were grouped according to their expression pattern. Different color bars depicted different clusters.

The analysis of K-means clustering of the 2,000 most variable genes across all samples is visually depicted in Figure 4. To better understand the functional implications, we identified enriched GO biological process pathways for each cluster, summarized in Table 1. The key findings from the clustering analysis are: Cluster B: during early lay, there was a robust effect of cell cycle and cell division. These effects were reduced as hens reached peak lay and continued to late lay. Cluster C: pathways related to ion transport and fibroblast growth factor were significantly enriched during peak lay, particularly in the sham group. Cluster D: pathways associated with cell communication, signaling, and response to stimulus showed increased activity during peak lay and late lay, notably in the sham groups. The identification of these dynamic gene expression pathways provides insights into the underlying molecular mechanisms that influence avian reproduction throughout the stages of production.

**TABLE 1:** Enriched gene ontology (GO) terms for biological process of K-means clustering with their significant values and number of genes presented in each pathway.

Cluster	Pathways	nGenes	adj.Pval
A	G protein-coupled receptor signaling pathway	31	2.1E-03
	Morphogenesis of an epithelial bud	5	2.3E-03
	Morphogenesis of an epithelial fold	5	3.0E-03
	Prostate gland morphogenesis	5	4.0E-03
B	Cell cycle process	55	6.8E-10
	Mitotic cell cycle process	42	6.8E-10
	Nuclear chromosome segregation	26	7.0E-10
	Cell cycle	68	8.2E-10
	Chromosome segregation	27	1.6E-09
	Mitotic cell cycle	45	2.6E-09
	Nuclear division	30	6.6E-09
	Mitotic nuclear division	25	9.9E-09
	Organelle fission	30	8.3E-08
	Mitotic sister chromatid segregation	18	1.1E-07
	Sister chromatid segregation	19	4.8E-07
	Attachment of spindle microtubules to kinetochore	7	1.4E-04
	DNA-dependent DNA replication	13	2.3E-04
	DNA replication initiation	7	2.3E-04
	Centromere complex assembly	6	1.1E-03
C	Ion transport	37	1.1E-05
	Transport	63	1.3E-03
	Cation transport	26	1.3E-03
	Ion transmembrane transport	26	1.3E-03
	Establishment of localization	64	1.3E-03
	Transmembrane transport	34	1.3E-03
	Fibroblast growth factor receptor signaling pathway	7	1.6E-03
	Cellular response to fibroblast growth factor stimulus	7	2.3E-03
	Response to fibroblast growth factor	7	2.3E-03
	Ureteric bud development	6	2.5E-03
	Mesonephros development	6	2.5E-03
	Mesonephric epithelium development	6	2.5E-03
	Mesonephric tubule development	6	2.5E-03
	Kidney morphogenesis	5	4.6E-03
	Sodium ion transport	8	5.6E-03
D	Ion transport	43	7.2E-04
	Multicellular organismal process	94	7.2E-04
	G protein-coupled receptor signaling pathway	28	9.9E-04
	Transmembrane transport	46	9.9E-04
	Cell communication	104	1.6E-03
	Signaling	103	1.6E-03
	Response to stimulus	126	3.5E-03
	Signal transduction	94	5.7E-03
	Regulation of membrane potential	12	5.7E-03
	Sulfate assimilation	3	8.9E-03

### 3.3 The presence of sperm alters UVJ transcriptome during peak lay and late lay

**FIGURE 5:**
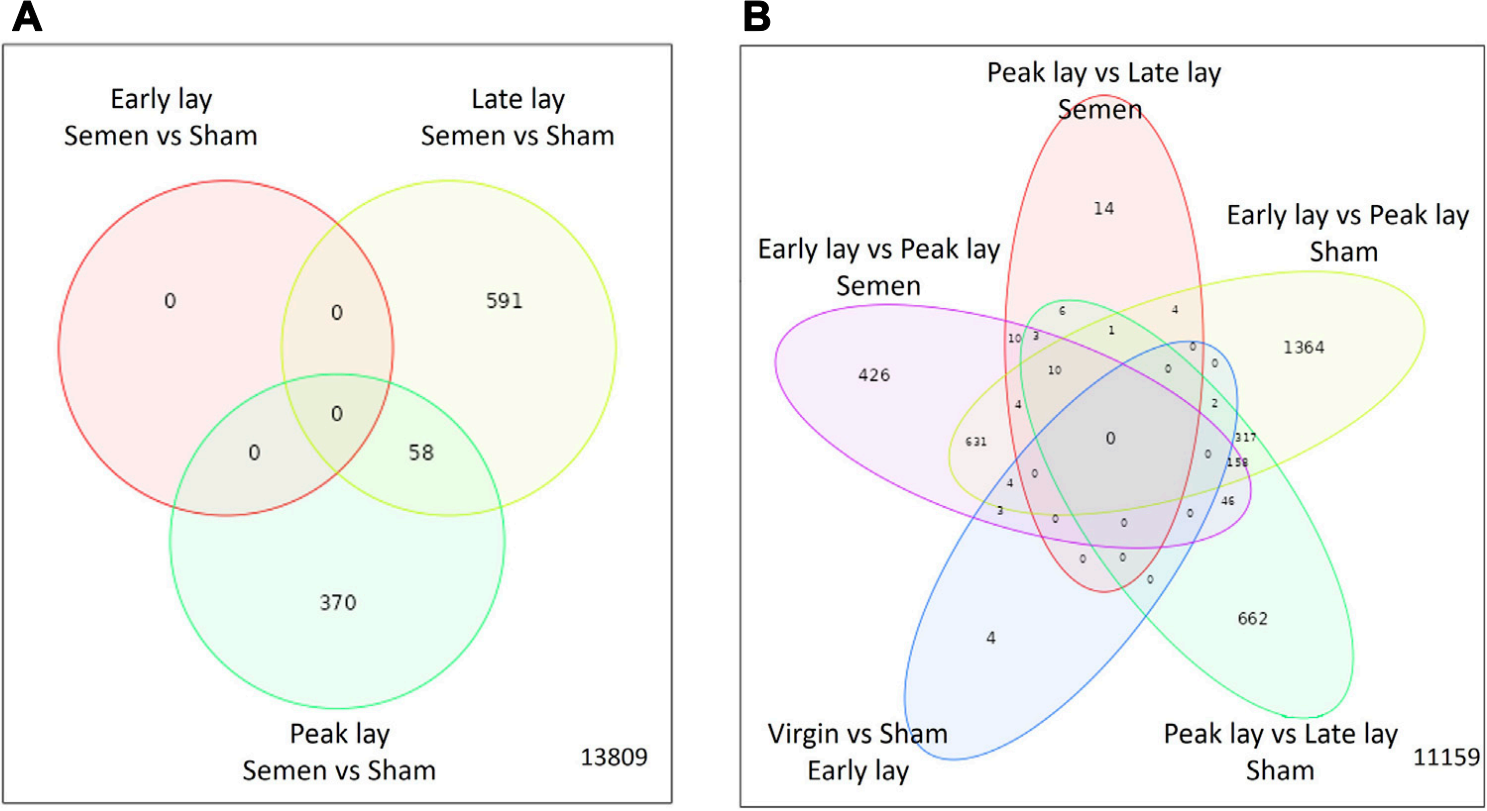
Characterization of differentially expressed genes in the UVJ. Venn diagrams show the number of common and unique differentially expressed genes in the semen vs. sham comparison at early lay, peak lay, and late lay **(A)** and among production stages comparisons in semen- and sham-inseminated groups **(B)**.

To investigate the influence of sperm on the UVJ transcriptome across the stages of production, we identified DEGs in the comparison between semen-inseminated and sham-inseminated birds (Supplementary Table S1). The number of DEGs identified at a threshold of FDR < 0.05 and fold-change > |1.5| between semen and sham birds across the stages of production are given in Table 2. As shown in Figure 5A, DEGs between semen and sham birds at peak lay and late lay were relatively unique, with limited overlapping genes, indicating distinct responses of the UVJ to sperm in each production stage. However, no significant expression changes were observed between semen and sham at early lay.

**TABLE 2:** Summary of gene expression and differential expressed genes (DEGs) of the UVJ between semen- and sham-insemination and across stages of production at FDR < 0.05 and fold-change > |1.5|.

Comparison	Groups	Total expressed genes	Common genes	Unique genes by group	DEGs	Up/Down genes
Treatment (Semen vs. sham)	Early lay	15,395	14,888	282/225	0	0/0
	Peak lay	15,252	14,661	288/303	428	225/203
	Late lay	15,242	14,647	232/363	649	464/185
Production stage						
Virgin vs. early lay	Sham	15,433	14,898	263/272	13	5/8
Early lay vs. peak lay	Sham	15,426	14,693	477/256	2,495	1,615/880
	Semen	15,367‬	14,710	403/254	1,295	895/400
Peak lay vs. late lay	Sham	15,251	14,577	372/302	1,205	546/659
	Semen	15,288	14,686	278/324	52	16/36

**FIGURE 6:**
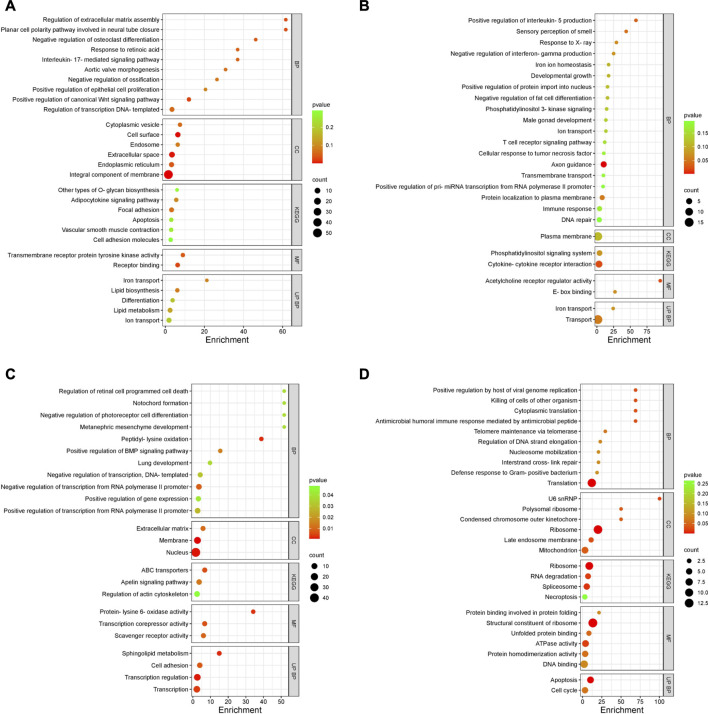
Enriched Gene Ontology (GO) terms of DEGs in the uterovaginal junction between semen vs. sham at peak lay and late lay. An enrichment bubble plot of upregulated **(A,C)** and downregulated **(B,D)** DEGs in semen group at peak lay and late lay, respectively. *P* values are represented by colors, gene counts are represented by bubble size. Abbreviations: BP, Biological process; CC, Cellular component; KEGG, Kyoto Encyclopedia of Genes and Genomes; MF, Molecular function; UP BP, UniProt biological process. Bubble plots were plotted by https://www.bioinformatics.com.cn/en, a free online platform for data analysis and visualization.

The enriched GO terms, KEGG pathways, and UniProt Knowledgebase (UniPortKB) keywords of DEGs between semen and sham comparison at peak lay and late lay are shown in Figure 6. Notably enrichment analysis of the DEGs during peak lay, found the ion transport pathway was enriched, with some genes being upregulated in the semen group and others downregulated (Figures 6A, B). Additionally, lipid biosynthesis and metabolism, Wnt signaling pathway, regulation of epithelial cell proliferation, and O-glycan biosynthesis were enriched and upregulated (Figure 6A). In contrast, the immune response and cytokine-cytokine receptor interaction were enriched but downregulated in semen-inseminated birds at peak lay (Figure 6B). Noteworthy, pro-inflammatory cytokine receptors including interleukin 1 receptor like 1 (*IL1RL1*), C-C motif chemokine receptor 7 (*CCR7*), tumor necrosis factor receptor superfamily member 6b (*TNFRSF6B*), Fas cell surface death receptor (*FAS*), and prolactin receptor (*PRLR*) were significantly downregulated in the semen-inseminated hens at peak lay (Supplementary Table S1).

**FIGURE 7:**
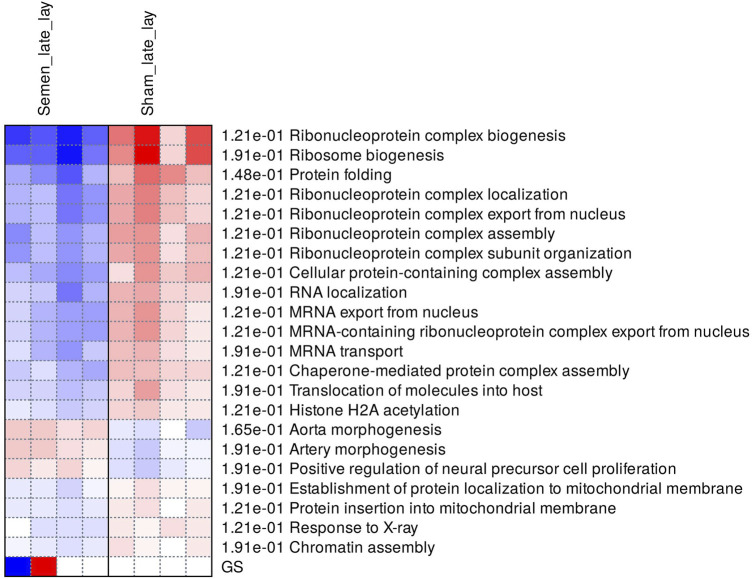
Enriched pathways of differentially expressed genes between semen- and sham-inseminated birds at late lay. A heatmap visualization of the top 30 enriched pathways reveal numerous ribosome-related and translation pathways in a downregulated direction in the semen group. Red and blue indicate activated and suppressed pathways, respectively.

**FIGURE 8:**
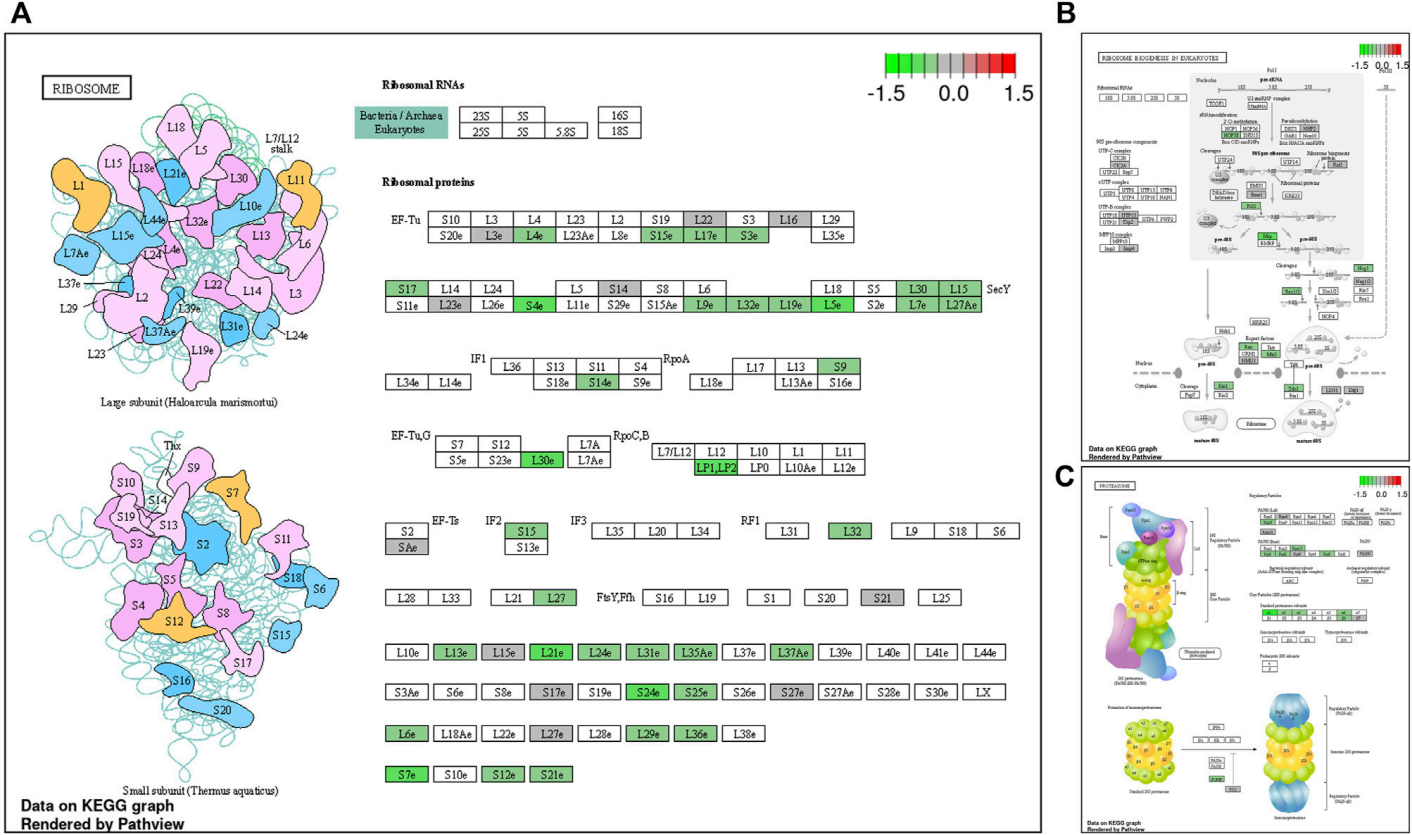
Enriched Kyoto Encyclopedia of Genes and Genomes (KEGG) pathway diagrams of downregulated differentially expressed genes between semen- and sham-inseminated birds at late lay. KEGG pathway diagrams illustrate downregulation of genes in the ribosome **(A)** ribosome biogenesis **(B)**, proteasome **(C)** pathways in the semen group at late lay. Fold-change (log2) cutoff in color code: bright red indicates most upregulated, bright green, most downregulated.

**FIGURE 9:**
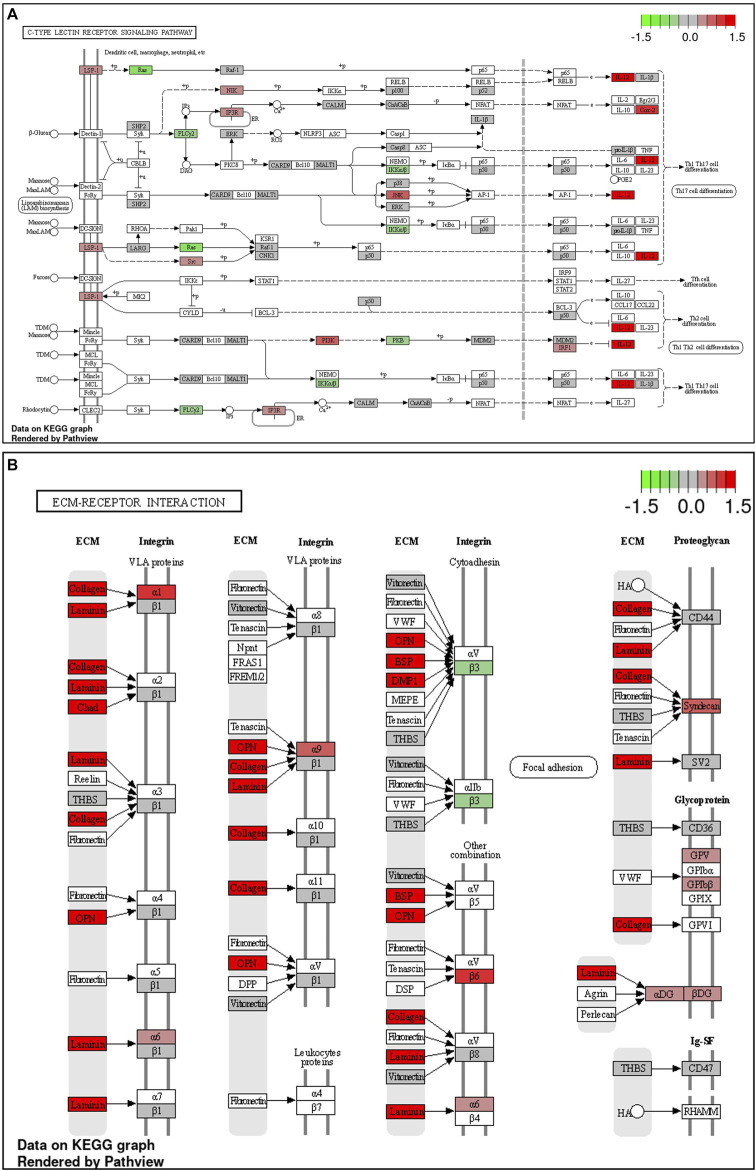
Enriched Kyoto Encyclopedia of Genes and Genomes (KEGG) pathways of upregulated differentially expressed genes between semen- and sham-inseminated birds at late lay. KEGG pathway diagrams illustrate upregulation of genes in the C-type lectin receptor signaling **(A)** and extracellular matrix (ECM)-receptor interaction **(B)** pathways in the semen group at late lay. Fold-change (log2) cutoff in color code: bright red indicates most upregulated, bright green, most downregulated.

During late lay, the UVJ exhibited an imbalance between transcription and translation. Specifically, genes upregulated in the semen group were enriched for GO terms related to transcription, while downregulated genes were enriched for GO terms related to translation compared to the sham group. The PGSEA analysis of the top 30 pathways (Figure 7) revealed significant downregulation in pathways associated with translation, protein synthesis, and modification in the semen group at late lay, compared to the sham group during the same stage of production. Additionally, telomere maintenance *via* telomerase and the cell cycle were enriched and downregulated in the semen group (Figure 6D). Genes related to the ribosome, ribosome biogenesis, and proteasome were downregulated in the semen group (Figures 8A–C). In contrast, the C-type lectin receptor signaling pathway (Figure 9A) and the extracellular matrix (ECM)-receptor interaction pathways (Figure 9B) were enriched and upregulated in the semen group compared to the sham group during late lay.

### 3.4 Transcriptomic alterations in the UVJ across the stages of production

To explore the transcriptomic changes throughout the production stages, we conducted a total of five pairwise comparisons. Specifically, we evaluated the transcriptomic alterations between early lay and peak lay, and between peak lay and late lay, in both semen and sham groups. Additionally, we made a comparison between virgin and sham groups at early lay. Table 2 summarizes the total number of DEGs from each pairwise comparison at different stages of production.

During the transition from early lay to peak lay, numerous DEGs were shared between the semen and sham groups. These genes are associated with reproductive development from early to peak lay, irrespective of the presence of sperm (Figure 5B). The sham groups exhibited the greatest number of DEGs during this transition, followed by the semen groups. A drastic decrease in DEGs was observed in semen groups from peak lay to late lay when compared to sham groups during the same period. Moreover, only a small number of genes were differentially expressed when comparing virgin and sham at early lay (Table 2).

Next, we examined the enriched GO terms for biological processes in the sham group during the transition from early lay to peak lay. This analysis revealed a decline in processes related to cell cycle, cell division, extracellular matrix organization, and cell adhesion, along with an increase in ion and transmembrane transports, cellular, ion, and chemical homeostasis, and endoplasmic reticulum stress (Table 3). Similar GO terms were enriched in the downregulated genes observed in the semen group during the same transition period.

**TABLE 3:** Enriched pathways of differentially expressed genes of sham-inseminated hens at peak lay compared to early lay.

Direction	Pathways	nGenes	adj.Pval
Downregulated	Extracellular structure organization	26	3.8e-05
	Extracellular matrix organization	25	4.2e-05
	External encapsulating structure organization	25	4.2e-05
	Cell cycle	93	2.2e-04
	Attachment of spindle microtubules to kinetochore	9	3.3e-04
	Cell cycle process	69	4.4e-04
	Nuclear chromosome segregation	27	4.4e-04
	Mitotic cell cycle	56	5.4e-04
	Nuclear division	34	8.8e-04
	Microtubule-based process	67	9.3e-04
	Chromosome segregation	28	1.0e-03
	Cell adhesion	78	1.0e-03
	Biological adhesion	78	1.0e-03
	Organelle fission	36	1.0e-03
	Movement of cell or subcellular component	103	2.1e-03
Upregulated	Response to endoplasmic reticulum stress	23	5.7e-06
	Ion transport	77	4.2e-05
	Protein targeting to ER	9	4.2e-05
	Transport	165	1.4e-04
	Establishment of localization	169	1.4e-04
	Establishment of protein localization to endoplasmic reticulum	9	1.4e-04
	Inorganic ion homeostasis	30	1.7e-04
	Ion homeostasis	31	2.6e-04
	Cation homeostasis	29	2.6e-04
	Cellular homeostasis	32	4.7e-04
	Cellular ion homeostasis	25	6.4e-04
	Chemical homeostasis	39	6.4e-04
	Cellular chemical homeostasis	29	6.4e-04
	Transmembrane transport	79	6.4e-04
	Protein localization to endoplasmic reticulum	9	6.4e-04

At late lay, the enriched GO terms in the sham group indicated an upregulation of processes related to microtubule-based movement and transport, along with cilium assembly, organization, and movement (Table 4). Conversely, the nucleoside biphosphate and thioester metabolic processes were enriched and downregulated in the semen group at late lay. These findings provide insights into the dynamic changes in gene expression patterns and shed light on the biological processes and pathways involved in the UVJ during different stages of production.

**TABLE 4:** Enriched pathways of differentially expressed genes of sham-inseminated hens at late lay compared to peak lay.

Direction	Pathways	nGenes	adj.Pval
Upregulated	Microtubule-based process	52	5.4e-11
	Cilium organization	30	1.6e-10
	Microtubule-based movement	29	1.2e-08
	Cell projection assembly	31	1.2e-08
	Cilium assembly	26	1.2e-08
	Cilium movement	17	1.2e-08
	Axoneme assembly	12	3.8e-08
	Plasma membrane bounded cell projection assembly	30	3.8e-08
	Cell projection organization	50	6.1e-07
	Microtubule cytoskeleton organization	31	6.1e-07
	Microtubule bundle formation	12	7.6e-07
	Organelle assembly	35	1.6e-06
	Plasma membrane bounded cell projection organization	47	2.9e-06
	Microtubule-based transport	13	6.9e-05
	Determination of left/right symmetry	11	7.8e-05

## 4 Discussion

In this study, we investigated the transcriptomic changes in the UVJ in response to repeated insemination throughout the production cycle of female turkey breeders. Our objective was to identify the factors contributing to declining fertility following prolonged and repeated insemination as hens advanced through their production cycles. It is important to note that our study did not include virgin controls at each time point, which represents a limitation in our experimental design. The conclusions and discussions drawn were primarily grounded in the comparison between semen-inseminated and sham-inseminated groups, both within various stages of production and across different production stages. In our study, we found that the transcriptome of the UVJ undergoes dynamic remodeling at the beginning of laying period. Robust gene expression mapping to the processes of cell cycle and cell division were dominant in early lay groups (virgin, sham, and semen), while these processes declined as hens progressed towards peak lay and remained suppressed during late lay. This suggests that the UVJ tissue undergoes essential modifications at the start of the laying period to prepare for forthcoming active egg-laying. Additionally, once hens entered their peak production phase, we observed a significant upregulation in ion transport and epithelium development in the UVJ. These processes appear to exert their roles during the peak lay phase of egg production. Ion transport is crucial for maintaining the ionic balance and proper functioning of the reproductive tract (Atikuzzaman et al., 2015; Yang et al., 2021a). Epithelium development is likely involved in optimizing the structure and function of the UVJ to support the high reproductive demands during peak production (Yang et al., 2023).

Although previous evidence indicated that the UVJ responds to sperm as early as 24 h after mating (Atikuzzaman et al., 2015), our study did not observe significant transcriptomic alterations in the UVJ between semen and sham groups at 48 h after the first insemination. The lack of changes could be attributed to species divergence, experimental conditions, and differences in technology used. However, we did observe a substantial number of genes differentially expressed in sham birds from early lay to peak lay. Notable effects in cell cycle process, cell division, and extracellular matrix organization during early lay demonstrate the development of the reproductive tract in preparation for the approaching increase in reproductive performance. We observed upregulation of genes in ion and transmembrane transport pathways of sham-inseminated hens entering peak lay. Interestingly, these favorable arrangements occurred naturally alongside the production cycle, regardless of the presence of sperm. We speculate that changes in reproductive hormones across the production stages contribute to the fundamental transformation of the UVJ tissue, and the presence of sperm further enhances tissue modification.

The role of fatty acids in sperm survival in the SST has been reported for the hen oviduct (Huang et al., 2016). Lipid synthesis and metabolism are altered in the transcriptome of the avian UVJ suggesting a role of sperm maintenance in the SST (Han et al., 2019; Yang et al., 2021a; Yang et al., 2021b; Brady et al., 2022). In our study, we observed upregulation of lipid biosynthesis and metabolism in the semen-inseminated birds compared to sham-inseminated birds during peak lay, including key genes such as 1-acylglycerol-3-phosphate O-acyltransferase 2 (*AGPAT2*), acyl-CoA synthetase bubblegum family member 2 (*ACSBG2*), diacylglycerol kinase beta (*DGKG*), ELOVL fatty acid elongase 2 (*ELOVL2*), fatty acid 2-hydroxylase (*FA2H*), and lipase G, endothelial type (*LIPG*) (Supplementary Table S1). The transition in lipid biosynthesis and metabolism of the UVJ during peak lay was triggered by the presence of sperm and seminal plasma, as the levels of these genes did not increase in sham comparisons between early lay and peak lay. Notably, a prominent upregulation of *ELOVL2* in semen-inseminated birds at peak lay perhaps marks a crucial role of its function in fertility. ELOVL2 catalyzes the first and rate-limiting reaction that constitutes the long-chain fatty acid elongation cycle (Zhang et al., 2016). Although studies on its function in female reproduction are lacking, several studies have demonstrated its fundamental role in male fertility in mammals (Zadravec et al., 2011; Olia Bagheri et al., 2021; Castellini et al., 2022; Liu et al., 2022).

Regulation of pH also plays a role in sperm survival and activity in the UVJ of birds (Holm et al., 1996; Holm and Wishart, 1998). In our studies, we observed an increase in *CA4* expression from virgin to early lay to peak lay in both sham and semen groups (Supplementary Table S2). The level further elevated only in the semen group at late lay. Carbonic anhydrases (CA) have long been studied for their roles in sperm motility and fertility in both male and female reproductive tracts of mammals and birds (Harris and Goto, 1984; Holm et al., 1996; Holm et al., 2000; Wandernoth et al., 2010; Wandernoth et al., 2015). We believe that CA4 plays a fundamental role in turkey UVJ, but its levels may not directly reflect fertility rates.

The avian innate immune system plays a dual role in the reproductive tract. It protects against microbial challenges, and at the same time, it establishes tolerance for sperm to support successful reproduction. In our study, we observed both immune activation and suppression pathways during the comparison of semen and sham groups at peak lay. An upregulation of antimicrobial peptides, including beta-defensin 2 (*THP2*) and cathelicidin-3 (*LOC104911326*) were noted in the semen group at peak lay (Supplementary Table S1). Antimicrobial peptides play a crucial role in protecting the reproductive tract from potential microbial threats. Simultaneously, we observed a downregulation of several pro-inflammatory cytokine and chemokine receptors in the semen-inseminated hens at peak lay. Pro-inflammatory cytokines and chemokines are essential in initiating immune responses and local inflammatory responses (Staeheli et al., 2001). These down-regulations appear to counterbalance the potential increase in cytokines and chemokines, aiming to attenuate local inflammation that could be triggered by foreign bodies, such as sperm. The suppression of the UVJ immune response is essential for sperm survival and storage, as it helps create a favorable environment for sperm within the reproductive tract. The decrease in cytokine-cytokine receptor interaction during peak lay may contribute to the improved survival and storage of sperm, ultimately reflecting a higher reproductive performance of the hen during this phase of the production cycle. However, as the production cycle progresses towards late lay, the expression pattern of these genes is reversed. We observed an upregulation of genes in the same immune families between semen groups at late lay compared to peak lay, indicating a rise in UVJ immune responses to sperm. This change was not observed in comparison to sham groups during the same time periods, illustrating the variation in local immune responses to sperm across different stages of production. These immune responses could be a crucial factor in determining the reproductive efficiency of turkey hens.

Several studies have reported an increase in transforming growth factor β and its receptor expression after insemination in avian species (Das et al., 2006; Atikuzzaman et al., 2015; Yang et al., 2023), suggesting a positive correlation of their expression with fertility (Gu et al., 2017). We also found an increase in *TGFB2* and its receptor in the semen group at peak lay with lower expression at late lay. Previous studies have suggested that TGF-β may suppress anti-sperm immune responses and protect sperm in the SST in chickens (Das et al., 2006). There is also evidence that TGF-β maintains tolerance to sperm cells in the murine epididymis (Voisin et al., 2020). Further investigation of TGF-β roles in the avian UVJ could enhance our understanding of sperm tolerance and the evasion of immune responses by the female reproductive tract.

In aging brains of mice and fish, a progressive reduction in the correlation between protein and mRNA levels is noted, often attributed to reduced proteasome activity (Kelmer Sacramento et al., 2020). Similar findings in roundworms and yeast indicate age-related degradation of ribosome function, leading to impaired protein synthesis and disrupted cellular processes (Stein et al., 2022). The transcriptomic analysis of semen-inseminated hens during late lay revealed a distinct pattern: an increase in transcription but a decrease in translation processes, indicating a potential reduction in overall protein synthesis. This pattern is reminiscent of a characteristic of aging, where there is often an observed loss of protein homeostasis. A downregulation of genes related to ribosomes, ribosome biogenesis, the proteasome, and protein export pathways observed in the UVJ of semen-inseminated hens at late lay implies the UVJ experiences advanced aging-like impairments, resembling the characteristics of aging in terms of altered protein synthesis, similar to what has been observed in the brains of aging mice and fish, which did not occur in the sham birds at the same production stage. These findings indicate that the UVJ exposed to repeated insemination exhibits characteristics of advanced aging, including loss of organelle function and telomere maintenance. Several recent studies have demonstrated an association of ribosome disruption and aging, adding to the relevance of our findings (Gonskikh and Polacek, 2017; Turi et al., 2019; Skariah and Todd, 2021; Stein et al., 2022).

Taken together, our study highlights that the transcriptome of the turkey UVJ undergoes dynamic alterations in response to laying status and the frequency and duration of semen exposure. Tissue remodeling occurs at the start of lay, preparing the UVJ to function as a sperm storage site. Local immune suppression appears to promote sperm survival and storage during peak lay, which coincides with the peak fertility period. At the late production stage, when hens had experienced prolonged repeated insemination, the UVJ exhibited a loss of protein homeostasis, resembling aging-like impairment. This was accompanied by an increase in immune responses, which could potentially lead to a loss of proper UVJ function and a favorable environment for sperm storage. To further explore possibilities to prevent or delay the destruction of the UVJ, we plan to investigate immune responses and reproductive aging at the single-cell level, aiming to gain deeper insights into the underlying mechanisms responsible for these phenomena. Understanding these mechanisms may provide potential strategies to maintain the UVJ’s functionality and slow fertility decline in turkey hens.

## Data Availability

The datasets presented in this study can be found in online repositories. The names of the repository/repositories and accession number(s) can be found below: (NCBI) BioProject: PRJNA1003625.

## Supplementary material

SUPPLEMENTARY TABLE S1 Summary of differentially expressed genes of semen and sham comparison across production cycle and among production cycle in semen and sham groups.

SUPPLEMENTARY TABLE S2 Mean quality-trimmed RNAseq read counts of UVJ tissues from virgin birds, sham- and semen-inseminated birds sampling at early lay, peak lay, and late lay.
